# A Research Agenda for Helminth Diseases of Humans: Intervention for Control and Elimination

**DOI:** 10.1371/journal.pntd.0001549

**Published:** 2012-04-24

**Authors:** Roger K. Prichard, María-Gloria Basáñez, Boakye A. Boatin, James S. McCarthy, Héctor H. García, Guo-Jing Yang, Banchob Sripa, Sara Lustigman

**Affiliations:** 1 Institute of Parasitology, McGill University, Montreal, Canada; 2 Department of Infectious Disease Epidemiology, School of Public Health, Faculty of Medicine, Imperial College London, London, United Kingdom; 3 Lymphatic Filariasis Support Centre, Department of Parasitology, Noguchi Memorial Institute for Medical Research, University of Ghana, Legon, Ghana; 4 Queensland Institute of Medical Research, University of Queensland, Herston, Australia; 5 Department of Microbiology, Universidad Peruana Cayetano Heredia, Lima, Peru; 6 Department of Schistosomiasis Control, Jiangsu Institute of Parasitic Diseases, Meiyuan Yangxiang, Wuxi, People's Republic of China; 7 Tropical Disease Research Laboratory, Division of Experimental Pathology, Department of Pathology, Khon Kaen University, Khon Kaen, Thailand; 8 Laboratory of Molecular Parasitology, Lindsley F. Kimball Research Institute, New York Blood Center, New York, New York, United States of America; London School of Hygiene & Tropical Medicine, United Kingdom

## Abstract

Recognising the burden helminth infections impose on human populations, and particularly the poor, major intervention programmes have been launched to control onchocerciasis, lymphatic filariasis, soil-transmitted helminthiases, schistosomiasis, and cysticercosis. The Disease Reference Group on Helminth Infections (DRG4), established in 2009 by the Special Programme for Research and Training in Tropical Diseases (TDR), was given the mandate to review helminthiases research and identify research priorities and gaps. A summary of current helminth control initiatives is presented and available tools are described. Most of these programmes are highly dependent on mass drug administration (MDA) of anthelmintic drugs (donated or available at low cost) and require annual or biannual treatment of large numbers of at-risk populations, over prolonged periods of time. The continuation of prolonged MDA with a limited number of anthelmintics greatly increases the probability that drug resistance will develop, which would raise serious problems for continuation of control and the achievement of elimination. Most initiatives have focussed on a single type of helminth infection, but recognition of co-endemicity and polyparasitism is leading to more integration of control. An understanding of the implications of control integration for implementation, treatment coverage, combination of pharmaceuticals, and monitoring is needed. To achieve the goals of morbidity reduction or elimination of infection, novel tools need to be developed, including more efficacious drugs, vaccines, and/or antivectorial agents, new diagnostics for infection and assessment of drug efficacy, and markers for possible anthelmintic resistance. In addition, there is a need for the development of new formulations of some existing anthelmintics (e.g., paediatric formulations). To achieve ultimate elimination of helminth parasites, treatments for the above mentioned helminthiases, and for taeniasis and food-borne trematodiases, will need to be integrated with monitoring, education, sanitation, access to health services, and where appropriate, vector control or reduction of the parasite reservoir in alternative hosts. Based on an analysis of current knowledge gaps and identification of priorities, a research and development agenda for intervention tools considered necessary for control and elimination of human helminthiases is presented, and the challenges to be confronted are discussed.

## Introduction

The increasing recognition of the burden imposed by human helminthiases has led to the implementation of large-scale control and elimination programmes. In the accompanying review of this collection (“The Problem of Helminthiases”), Lustigman et al. [1] summarise the historical development and remit of such initiatives. In this paper, Table 1 presents the various programmes' aims and strategies, as well as the helminths they target. Although these programmes recognise the importance of ancillary strategies such as environmental improvement, increased hygiene, and sustained socioeconomic development, targeted mass drug administration (MDA) has become their mainstay. The drugs (anthelmintics) involved are in some cases donated by pharmaceutical companies (e.g., ivermectin [IVM] by Merck & Co. for onchocerciasis and lymphatic filariasis [LF]; albendazole [ABZ] by GlaxoSmithKline for LF, and soil-transmitted helminthiases [STHs] in Africa; mebendazole [MBZ] for STHs, by Johnson & Johnson), and in other cases are affordable as generic preparations (e.g., praziquantel [PZQ] for schistosomiasis; diethylcarbamazine [DEC] for LF). In general, the anthelmintic drugs adopted by the control programmes are administered as a single dose at regular intervals, generally yearly or twice yearly. Used in this way, they are usually safe for mass treatment of human populations and are moderately effective in terms of reducing the intensity of infection and, in some cases, curing a proportion of infected individuals.

**Table 1:** Major Mass Drug Administration Programmes to Control Helminth Diseases of Humans.

Infection	Causal Agent(s)	Number Infected (Millions)	DALYs (Millions)	Programme	Main Objective	Strategy	Timescale
Onchocerciasis	*Onchocerca volvulus*	37	1.5	OCP	Elimination of morbidity	Initially vector control; after 1988 annual IVM (CDTI)	1974–2002
				National OCPs - W. Africa	Continue from OCP	IVM CDTI	2002–no date set
				OEPA	Elimination of parasite	IVM MDA every 6 months	1992–2007–2012
				APOC	Elimination of morbidity (& parasite where possible)	IVM CDTI in meso- and hyperendemic areas	1995–2009–2015–2025
Lymphatic filariasis	*Wuchereria bancrofti; Brugia malayi; B. timori*	120	5.8	GPELF	Elimination of parasite	5 or more years of annual IVM+ABZ (sub-Saharan Africa); DEC+ABZ or DEC (rest of world)	1999–2020
Soil-transmitted helminths	*Necator americanus; Ancylostoma duodenale; Ascaris lumbricoides; Trichuris trichuria*	>1,000	9.4–39	PPC, DtW, GPELF, SCI	Control of morbidity	ABZ or MBZ 1–3 times/year	2001–no date set
Schistosomiasis	*Schistosoma mansoni; S. haematobium; S. japonicum*	207	1.7–4.5	JRMC, SCI	Control of morbidity	Treat >75% school-aged children & other high risk groups annually with PZQ. Integrated intervention (humans, reservoir hosts, snails) for *S. japonicum*	JRMC: 1992–1999; SCI: 2000–no date set
Clonorchiasis, opisthorchiasis	*Clonorchis sinensis*, *Opisthorchis viverrini*, *Op. felineus*	56	0.5–0.9	National	Control of morbidity	PZQ annually/biennially	2011–no date set

Adapted from [1], [64], [105], [137,138].

OCP, Onchocerciasis Control Programme in West Africa (after closure of OCP, national governments continue control); OEPA, Onchocerciasis Elimination Program for the Americas; APOC, African Programme for Onchocerciasis Control; GPELF, Global Programme to Eliminate Lymphatic Filariasis; PPC, Partners for Parasite Control; DtW, Deworm the World; SCI, Schistosomiasis Control Initiative; JRMC, Joint Research Management Committee, China; CDTI, Community Directed Treatment with Ivermectin (IVM); ABZ, albendazole; DEC, diethylcarbamazine; MBZ, mebendazole; PZQ, praziquantel.

In the last decade, epidemiological studies in areas under control have demonstrated successes in addressing human helminthiases. The latter include infections by nematodes (roundworms), and trematodes and cestodes (flatworms). Among the roundworms, this review focuses on filarial infections (particularly those caused by *Onchocerca volvulus* and *Wuchereria bancrofti*), as well as on the most common soil-transmitted helminth infections caused by *Ascaris lumbricoides* (roundworm), *Trichuris trichiura* (whipworm), *Necator americanus*, and *Ancylostoma duodenale* (hookworms). Among the trematode infections, we focus on those caused by *Schistosoma* species and the food-borne trematodiases, and among the cestode infections, we address some issues related to taeniasis and (neuro)cysticercosis (caused by *Taenia solium*).

However, mass chemotherapy as a control strategy has challenges, including optimising community involvement and participation, and monitoring and evaluating the effectiveness and impact of MDA. While global funding for these programmes has increased in recent years, enthusiasm tends to wane after the initial successes (which are invariably largest at the beginning), and “donor fatigue” becomes a risk. Other issues include the fact that the arsenal of available drugs is limited, with very little development of new drugs specifically targeted to human helminthiases. This makes the existing control programmes highly vulnerable should anthelmintic resistance develop and spread, as is possible following years of MDA [2], [3]. Billions of doses of anthelmintics have been administered to infected and exposed individuals, in some cases for prolonged periods, and have undoubtedly yielded health benefits for the treated populations. However, helminth infections persist in their host populations and show resilience to control. Some of the factors responsible for the persistence of human helminthiases as a public health problem, despite the many initiatives that have been launched for their control, are discussed by Lustigman et al. [1]

In this review, and based on deliberations by the Disease Reference Group on Helminth Infections (DRG4), established in 2009 by the Special Programme for Research and Training in Tropical Diseases (TDR), a summary of the current strategies and available tools for the control of single helminth infections is presented, followed by a discussion of the challenges posed by integrated control in the face of co-endemicity and polyparasitism with more than one infection. A research and development agenda is presented that addresses the need for novel intervention tools in addition to the employment of existing ones, as well as the requirement for monitoring and evaluation (M&E) of the interventions, the design of surveillance systems, and the development of better assays to detect promptly decreased intervention efficacy and emerging anthelmintic resistance. A summary of key points for successful intervention are presented in Box 1. This agenda and its challenges are discussed below in the context of morbidity control and elimination programmes.

**Box 1. Summary Points for Control Interventions against Helminthiases**

- Major intervention programmes have been launched against onchocerciasis, LF, STHs, and schistosomiasis, which are mostly dependent on MDA of anthelmintics and require annual or biannual treatment of large numbers of populations at risk. The implementation of these programmes has been aided by drug donation (ivermectin, albendazole, mebendazole), and drug affordability (diethylcarbamazine, praziquantel)
- Co-endemicity and polyparasitism are common and there are advantages in integrating intervention efforts for different diseases. This requires an understanding of the implications of integration of programmes, effects on treatment coverage, and combination of suitable pharmaceuticals
- The efficacy of current anthelmintics ranges from moderate to relatively high, and some drugs do not kill the adult worms but reduce reproduction and thus transmission
- Some programmes aim at reducing morbidity and transmission (preventative chemotherapy), whilst others aim at parasite elimination, requiring repeated treatments over many years to reduce or suppress environmental contamination or vector/intermediate host infection with transmission stages. The continued and prolonged MDA with the same limited number of anthelmintic monotherapies over many years greatly increases the probability that drug resistance will develop, raising concerns about the sustainability of control and the achievement of elimination
- For elimination of these diseases, there is a need for the development of: 1) better anthelmintics and new formulations (e.g., paediatric formulations); 2) additional control tools such as vaccines and their integration into current strategies; 3) complementation of MDA with vector control where possible; 4) new monitoring tools with which to detect low level infections that may play a role in maintaining transmission; 5) tools to understand and monitor anthelmintic resistance
- To achieve ultimate elimination of human helminthiases, MDA and individual drug treatments need to be integrated with education, sanitation, environmental improvement, adequate monitoring and surveillance, and other available and novel control tools

## Community and Individual Interventions

Although the goal of some programmes (Table 1) has been that of morbidity control and elimination of the public health burden (STHs, schistosomiasis, and onchocerciasis in Africa for the most part), others aim at interruption of transmission and eventual elimination of the parasite reservoir (LF, onchocerciasis through vector control/elimination in Africa, onchocerciasis in Latin America, dracunculiasis). However, most of the helminth diseases cause chronic and often sub-clinical morbidity. Therefore, intervention is often at the community level aimed at reducing both morbidity and transmission in the community. However, treated people are subject to reinfection when the environmental source or vector/reservoir is not sufficiently reduced. Further, as discussed below, the available drugs are not reliably curative. With community-based intervention, the expectation is that the impact of treatment will be sustainable because the burden of infective stages in the environment will be progressively reduced by repeated rounds of treatment.

Anthelmintic treatment can be aimed at particular occupational or age groups that are thought to represent those most at risk of acquiring heavy infection and subsequently developing severe morbidity. This is, for instance, the basis for school-based health programmes aimed at deworming children of STHs and schistosomiasis. In this target population, treatment is administered to all individuals regardless of whether or not they are patently infected. In areas of substantial endemicity, and according to infection prevalence thresholds, community (mass) treatment is recommended. Other strategies include mass screen and treat (targeting selective treatment to those with patent and detectable infection at the point of screening), or treating individual cases in clinical as opposed to community settings. The adoption of such treatment modalities may also depend on the stage of the control programme, with MDA implemented at its commencement, and selective treatment used in mopping-up phases.

Optimum treatment coverage with MDA is required for the success of control and elimination programmes for helminth infections. Mathematical models predict, and experience confirms, that population coverage is a key determinant of the success of such programmes. Thus, there is a need to evaluate the compliance of the population participating in such programmes over the years. The use of the term “therapeutic coverage” (the proportion of the population treated) implies that the persons who receive the drugs are actually taking them (i.e., they are compliant), but in some countries, a gap between coverage and compliance has been observed, as for instance in the case of LF [4]–[6], and there may be systematic non-compliance (with the same group of people failing to take the drugs). The contribution of non-compliant persons to transmission has not been quantified for most of the infections, but systematic non-compliance may represent a potential threat for helminth control or elimination. Furthermore, the sustainability of the required long-term treatment programmes also raises issues of compliance related to possible population fatigue, waning interest of community drug distributors, and continuing funding by external donors, as the original funds that initiated the treatment-based control programmes may be time-limited.

Anthelmintic treatment of symptomatic individuals with hookworm, schistosomiasis, or neurocysticercosis can have marked effects in reducing morbidity. Therefore, interventions for the control of STHs and schistosome infections involve also chemotherapy of individuals. The control of food-borne trematodiases is usually on an individual patient basis and not via large-scale MDA. However, MDA with triclabendazole has recently commenced for fasciolasis. The control of taeniasis/cysticercosis is undertaken on an individual basis following case detection, and more recently by MDA with niclosamide or PZQ. In addition, oxfendazole treatment of pigs, as intermediate hosts, complements human taeniasis treatment and can decrease the time to elimination [7], [8].

## Current Intervention Tools

### Vector Control

Control of the vectors (including snail hosts) has been partially successful for onchocerciasis, particularly in West Africa [9], schistosomiasis in China [10], and more locally in some LF foci (e.g., in Zanzibar [11], and India [12]) and can be a valuable adjunct to other forms of parasite control. However, vector control currently is not the primary intervention used against helminthiases, and we have considered it as beyond the scope of this review.

### Drugs

The majority of the control programmes listed in Table 1 rely heavily on anthelmintics, and overall have resulted in significant reductions in the prevalence and intensity of infection and morbidity in some endemic areas [13]–[16]. The drugs used for each targeted helminth infection are detailed below. Interruption of transmission for some infections, leading towards local elimination, has been reported [17]–[21]. One reason for this heavy reliance on chemotherapy is because the drugs are donated for some programmes by pharmaceutical companies, or their cost has become affordable [22]. This intervention has been referred to as “a rapid-impact package” because the impact of anthelmintic treatment on parasite populations is proportionally largest at the beginning of the intervention [23]. However, long-term sustainability of the benefits accrued will critically depend on altering the environmental components that facilitate transmission. Large-scale elimination of the infection reservoir will depend on improving sanitation and drainage, providing access to clean water, disposing adequately of excreta and solid waste, promoting access to health services for diagnosis and treatment, and facilitating adequate housing and health education [24], [25]. The issues concerning the environmental and social ecology of human helminthiases are discussed in the accompanying review by Gazzinelli et al. [26]. Moreover, in programmes where the objective is eliminating the infection reservoir, treatment will be aimed to include the largest number of people with the highest possible coverage for as long as autochthonous transmission persists. This may impose strong selection pressures upon parasite genomes, affecting genetic diversity and favouring drug-resistant strains.

## Current Control Programmes and Ongoing Challenges

### Filarial Infections

Tens of millions of people living in hyper- and mesoendemic areas in Africa and in hypoendemic areas in Latin America are receiving annual or semi-annual treatment for onchocerciasis with IVM, while hundreds of millions of people across the world are receiving annual treatment for LF. For onchocerciasis, the only drug available for safe mass treatment is IVM. For LF, mass chemotherapy uses DEC plus ABZ or DEC alone, outside of sub-Saharan Africa, where IVM plus ABZ is used because of contraindications for DEC in patients infected with *O. volvulus*. IVM is highly microfilaricidal and also suppresses adult worm reproduction (production of new microfilariae [mf]) for several months in onchocerciasis [27] and LF [28]. DEC is also highly microfilaricidal, suppresses adult worm reproduction for several months, and kills a fraction of the adult lymphatic filariae. ABZ appears to contribute to the suppression of adult worm reproduction [29]. It also has effects on STHs (see below), producing possible ancillary benefits in those treated. Vector abatement can also reduce transmission and support the control of onchocerciasis [30], [31] and LF [32], [33].

In Africa, drug distribution is delivered by a system named Community Directed Treatment with Ivermectin (CDTI). For the control of onchocerciasis and LF, intervention is largely dependent on MDA where communities at risk are treated in order to reduce morbidity and parasite transmission. In the case of LF, topical treatment of individuals with affected limbs or other organs with antiseptic washes should be used as an adjunct treatment to reduce morbidity [34]. These mass chemotherapy programmes aim to achieve a high level of coverage (65%–90%) of the eligible population. Coverage comprises two components, geographical coverage (percentage of communities treated in an endemic area) and therapeutic coverage (percentage of the population treated in a community). Individuals who are eligible but do not present for treatment are considered as non-compliant. In addition, individuals who present for treatment, are given the medication, but do not swallow (do not take the medication or spit it out) are also non-compliant. A recent study of treatment for LF in India found 30.6% of people given medication actually did not take the anthelmintics [35]. In another drug compliance study among adults it was revealed that although most long-term residents included in the study, in Uganda, had been offered treatment at least in the 4 years of the study, the actual take up of drugs (proportion of the population who are offered free drugs who actually ingest the medication) for schistosomiasis and STHs varied considerably (0%–100%) from one district to another and often also within districts [36]. The specific reasons why MDA succeeded in some locations and faltered in others related to local dynamics. Both of these studies found that issues such as population movement, changing food supply, relations between drug distributors and targeted groups, rumours and conspiracy theories about the “real” purpose of treatment, subjective experiences of side effects from treatment, alternative understandings of affliction, responses to social control measures, and historical experiences of public health control measures all made a huge difference. These papers highlight the need to adapt MDA to local circumstances. It also points to issues that can be generalised, notably with respect to health education, drug distribution, and more effective use of existing public health legislation. The authors concluded that current standard practices of monitoring, evaluation, and delivery of MDA for neglected tropical diseases (NTDs) are inconsistent and inadequate. Furthermore, efforts to integrate programmes can exacerbate the difficulties. Improved assessment of what is really happening on the ground is an essential step in achieving long-term overall reduction of the NTD burden for impoverished communities.

The use of IVM in areas where loiasis (caused by another filarial parasite, *Loa loa*) is co-endemic can be contra-indicated due to rare, yet severe adverse events (SAEs), including fatalities [37]. Not all people with heavy *L. loa* infections experience SAEs, and further research is needed to understand factors that predispose some individuals to SAEs [38], [39]. This knowledge is important not only to avoid the incidence of SAEs but also because MDA programmes using IVM (onchocerciasis and LF) are not being conducted in regions with heavy loiasis (see [40] for discussion).

Another notable gap is the lack of safety data and an appropriate formulation of IVM suitable for administration to children under 5 years of age and under 15 kg of body weight. The need for paediatric formulations of anthelmintics has recently been reviewed [41]. The ability to treat safely small children would allow community coverage to be increased in MDA programmes. In some areas, ongoing transmission of filarial infection has been shown to persist despite more than 20 years of MDA. In some settings this is likely due to sub-optimal treatment coverage leading to ongoing transmission; in others a failure to implement effective vector control measures has impeded control. Other factors include parasite genetic factors such as heterogeneity in drug susceptibility, and when this deteriorates with repeated treatment, the possible development of drug resistance. Priority research challenges therefore include the implementation of studies designed to understand the relative contributions of treatment coverage, compliance patterns, intensity of infection, intensity of transmission, and parasite genetics/drug resistance on the ability of individual hosts and their parasites to respond to treatment.

Mass treatment programmes for onchocerciasis and LF are dramatically reducing transmission and thus morbidity. In some foci of the Americas, Mali, Senegal, and Nigeria (Kaduna), encouraging evidence indicates that that elimination of onchocerciasis may be possible using MDA with IVM in settings when high levels of therapeutic and geographic coverage over many years are achieved (14 years in Mali and Senegal) [20], initial endemicity levels are low to moderate, and the competence of the local vector species is relatively low [42]. Similarly, there are encouraging data from studies reporting the results of LF elimination programmes in some parts of the world (e.g., Egypt) [16]. Nevertheless, more research is needed to evaluate the optimal treatment regimes to achieve elimination. For instance, in some foci, more frequent treatment than the standard strategy of annual or biannual MDA has led to a rapid suppression of transmission even in formerly hyperendemic communities [43].

Annual treatment of *O. volvulus* infection with IVM, although effective in reducing microfilarial load and morbidity, may not be the optimal treatment schedule for interruption of transmission and prevention of selection for IVM resistance compared with more frequent schedules such as biannual treatment, a protocol that was adopted by the Onchocerciasis Elimination Program for the Americas (OEPA) in Latin America. More frequent treatment further reduces microfilarial load and transmission to vectors, and may therefore reduce the fitness advantage conferred to female worms that return more rapidly to fertility after IVM treatment. By contrast, the logistics of providing biannual treatment, and its possible effect of increasing the rate of selection for resistance (the simple argument that more frequent treatment would increase the pressure to select for resistant worms), may mean that annual IVM treatment is a better option. These are important research questions yet to be addressed. Studies to address this issue need to be undertaken in more than one setting, firstly where no or few rounds of IVM treatment have been dispensed, and thus no existing selection for drug resistance has occurred, and secondly where there have been many rounds of IVM treatment and there is evidence of a sub-optimal response.

### Soil-Transmitted Helminthiases

Interventions for the control of STHs entail MDA and/or individual chemotherapy, as well as improved sanitation, environmental conditions, and education. MDA programmes for STHs are most often directed at school-aged children as they are the population group most at risk of acquiring heavy infection and developing associated morbidity, and are most accessible for intervention through school-based programmes. However, some MDA programmes are directed at whole communities in highly endemic areas. The scale of MDA interventions for STHs has increased significantly in recent years and active surveillance for adverse events is warranted, although most will be mild.

In contrast to the programmes for filariasis, the focus of MDA programmes for STHs is to achieve long-term reduction in infection prevalence and intensity, and consequently of the associated morbidity, rather than aiming for elimination of infection. The health outcomes aimed for in STH programmes are closely related to those of poverty reduction, and improvements in education and sanitation. The apparent limitations of MDA programmes based on single dose anthelmintic monotherapy for STHs have been highlighted recently [44]. Although single dose therapy with either ABZ or MBZ is currently highly effective against *A. lumbricoides*, this is not the case for hookworms and whipworms (Table 2). As highlighted in the review by Geary et al. [45], one priority research need is to undertake well designed dose-finding studies to optimise the dose regimes of existing drugs used for treatment of STHs. Recently, the genetic polymorphisms in β-tubulin, which cause benzimidazole resistance in livestock parasites, have been found in *T. trichiura* and *N. americanus*. Part of the cause of the low/variable efficacy of ABZ or MBZ in *T. trichiura* and *N. americanus* may be due to benzimidazole-resistant genotypes in these parasites in some populations [46]. This needs urgent investigation, as a lack of understanding on this issue may jeopardise the sustainability of control efforts against STHs. DNA-based assays to detect the occurrence of these single nucleotide polymorphisms (SNPs) have been developed so that resistance-associated SNPs can be monitored in stool samples of STH eggs [47]. Routine M&E should be essential components for all deworming programmes.

**Table 2:** Cure Rates (%, Mean (Range)) of Anthelmintics against Soil-Transmitted Helminths.

Drug	Dose	*Ancylostoma duodenale* and *Necator americanus* (hookworms)^a^	*Trichuris trichiura* (whipworm)	*Ascaris lumbricoides* (roundworm)	*Enterobius vermicularis* (pinworm)	*Strongyloides stercoralis* (threadworm)
Albendazole	400 mg	72(59–81)	28(13–39)	88(79–93)	—(40–100^b^)	—(17–95^b^)
Mebendazole	500 mg	15(1–27)	36(16–51)	95(91–97)	96^b^—	44^b^—
Ivermectin	200 µg/kg	—(0–20^b^)	—(11–80^b^)	50–75^b^	—(61–94^b^)	—(83–100^b^)
Pyrantel	10 mg/kg	31(19–42)	—(0–56^b^)	88(79–93)	>90^b^—	—
Levamisole	2.5 mg/kg	—(66–100^b^)	—(16–18^b^)	86–100^b^	—	—

Adapted from [44] and b [139]. Means are available when meta-analysis of drug efficacy has been conducted and the figures in brackets are confidence intervals. Otherwise ranges are reported.

a *An. duodenale* is usually more susceptible to most anthelmintics than *N. americanus*.

b Indicates range or single value.

### Schistosomiasis

Interventions for the control of schistosome infections involve MDA and/or chemotherapy of individuals, as well as improved sanitation, environmental modifications to reduce exposure to the snail intermediate hosts and to cercariae that have been shed by snails, and education to reduce unsafe water contact. Schistosomiasis control programmes under the umbrella of the Schistosomiasis Control Initiative (SCI) (Table 1) are making progress towards the reduction of prevalence and intensity of infection and morbidity in Sub-Saharan Africa, with encouraging results from Mali, Burkina Faso, and Uganda [48]–[50]. In the latter, reductions in incidence have also been demonstrated, bringing benefits not only to those treated but also to those untreated members of the communities [21]. Spatial mapping of schistosomiasis has been significantly advanced in both West and East Africa [51], [52]. Operations research has highlighted the potential of lot quality assurance (LQA) sampling for identifying high risk communities for *S. mansoni* [53]. PZQ is virtually the only anti-schistosome medication used in MDA programmes. The development and thorough evaluation of a paediatric formulation of PZQ would allow greater population coverage in MDA programmes [54].

An interesting finding has been the recent demonstration that artemisinin-based compounds (e.g., artemether) are active against immature stages of schistosomes, which are relatively refractory to PZQ [55]. The artemisinins could prove useful should PZQ resistance become a problem. However, because the artemisinins are currently critically important for malaria chemotherapy (in light of resistance to most other antimalarials), artemisinins are not being used for anti-schistosome MDA. Nevertheless, experimental studies on the possible use of artemisinins, alone or in combination with PZQ, could be undertaken should concerns about selecting for artemisinin resistance in malaria diminish. Artemisinins (or an arteminisin-PZQ combination) could be developed as an arm of improved chemotherapy for schistosomiasis.

Although rodents can harbour *S. mansoni*, *S. haematobium* does not have non-human reservoirs, which facilitates control. Occasional infections with *S. bovis* in situations of very close proximity between humans and cattle have been reported [56]. *Schistosoma japonicum*, by contrast, is a zoonosis, and there have been important recent scientific advances in identifying which vertebrate species play important roles in transmission among themselves and to humans in China [57], [58] and the Philippines [59], [60]. Cattle and water buffalo may play a major role in the transmission of *S. japonicum* to humans in China and the Philippines [61], [62]. In Samar Province in the Philippines, an association exists between the intensity of infection of dogs and cats and infection in humans. Treatment or vaccination of reservoir hosts (e.g., bovines) should be implemented as part of the control programmes. In fact, an anti-schistosome fecundity vaccine with an efficacy of 50%–90% is being tested for bovine vaccination [63].

### Food-Borne Liver, Lung, and Intestinal Flukes

Besides PZQ treatment of individuals infected with *Clonorchis sinensis*, *Opisthorchis viverrini*, *Op. felineus*, *Paragonimus* spp., *Fasciolopsis buski*, *Echinostoma* spp., *Heterophyes heterophyes*, and *Metagonimus yokogawai*, there are MDA programs, using PZQ, in East Asia for clonorchiasis and opisthorchiasis [64]. Triclabendazole is the drug of choice for fascioliasis [65]. Recognition that liver fluke infection, caused by *Op. viverrini*, can be a significant cause of hepatic cancer has been an important scientific advance [66]. The hepatobiliary changes caused by *Op. viverrini* usually reverse after PZQ chemotherapy. However, resolution of hepatobiliary disease does not occur in all persons after the flukes have been eliminated. Problematically, these individuals may be at increased risk of cholangiocarcinoma [67]. In any event, upon parasitological diagnosis of liver fluke infection, immediate treatment with PZQ is indicated to eliminate long-lived, carcinogenic parasites [68]. A more aggressive education programme in Southeast Asia (to discourage consumption of raw fish and improved sanitary and food hygiene practices) could produce long-term reductions in liver fluke–induced morbidity and mortality. This will involve a greater recognition of the importance of these infections and greater efforts to prevent infection.

### Taeniasis and Cysticercosis

The control of taeniasis/cysticercosis is undertaken either by detection and treatment or MDA programmes. In addition, mass treatment of pigs as reservoir hosts of *T. solium* is being undertaken [69], [70]. Treatment of individuals with neurocysticercosis using a course of ABZ, over several days, kills parasites residing in the brain, but there is a risk when treating a viable brain cyst that inflammation could occur and trigger seizures [71] if not undertaken under appropriate medical supervision. Many people, in cysticercosis-endemic regions, are given MDA with ABZ or PZQ for other helminth infections, and there is a theoretical risk that these anthelmintics could precipitate seizures in individuals with occult neurocysticercosis. Prevention and control of *Taenia* infections should include enhanced sanitation and health education (to improve sanitary and food hygiene practices). These measures by themselves, however, are unlikely to be sufficient for control of taeniasis/(neuro)cysticercosis, and thus interventions including human chemotherapy, porcine chemotherapy, or porcine immunisation are required. Control of *Taenia* infections has greatly improved with the introduction of concomitant porcine mass chemotherapy using oxfendazole, and the development of TSOL18 as a highly effective porcine vaccine [72]. Proof of concept of the feasibility of actively eliminating *T. solium* transmission has also been provided in recent years in endemic areas of Peru and in Cameroon [69], [70]. The World Health Organization (WHO) has included taeniasis/cysticercosis in its Global Plan to combat Neglected Tropical Diseases 2008–2009, using new initiatives addressing integrated control of zoonotic diseases and in assessing the burden of food-borne disease.

## Co-Endemicity, Polyparasitism, and the Integration of Intervention Measures

Co-endemicity and polyparasitism are common in poor countries of the tropics and sub-tropics, the former meaning that a country or an area within a country (at a region or district level) can be endemic for more than one infection, and the latter meaning that the individual host/host populations may harbour more than one parasite species. This distinction is important for integration of control interventions at different geographical scales (from the country level to the community level), to determine the degree of true overlap of infections and the delineation of appropriately integrated control strategies [73]. Currently available single drug treatments can be integrated to target as many as four of the helminth NTDs, including onchocerciasis, LF, STHs, and schistosomiasis, by simultaneously administering for example, PZQ, ABZ, and IVM to communities where these helminth diseases may co-exist. In practice, different ecological requirements by the parasites and/or their vectors (e.g., fast flowing [lotic] water bodies for onchocerciasis as opposed to slow [lentic] water bodies for schistosomiasis) may translate into a lesser degree of overlap at the community level. Experience indicates that the co-administration of these three anthelmintics does not pose any significant concern of increased drug toxicity [65]. Nevertheless, there is evidence in both veterinary nematode parasites and *O. volvulus* that prolonged IVM treatment may select on the β-tubulin gene and increase the rate of selection for benzimidazole resistance [74]–[76]; thus, monitoring for resistance development should be undertaken in any integrated programmes that may be implemented. More operational research is needed to determine the best implementation strategies for providing drug therapies at regional and population levels [24], and to investigate the effects of integration on the achievement and maintenance of optimal coverage levels, treatment frequency, and treatment duration for each of the infections incorporated into the integrated strategy. Studies are required in the affected regions to identify practical methods to coordinate and execute the proposed treatment plans. Implementation of any helminth control programme at the country level will be best developed if strong links are created with existing interventions. An example of a process that can be implemented is to add anthelmintic treatment to well-established vaccination programmes. As there may be significant disparities between the age (or occupational) groups targeted for onchocerciasis, LF, STHs, and schistosomiasis, studies are necessary to identify optimal and common age groups for integrated control, where feasible. There is still the need to face additional political hurdles in order to facilitate groups that work with different diseases, and follow different protocols, to cooperate on disease control efforts and to fully integrate their activities.

The concept of integrating NTD management should go beyond anthelmintic chemotherapy as the only solution. As an example, Gray and coworkers [77] have pointed out that over US$350 million, until 2013, has been committed by donors for schistosomiasis control, relying almost exclusively on PZQ. However, chemotherapy by itself is not sufficiently effective, and sustainable donor funds should be spent on developing a multi-faceted, integrated programme of control, possibly involving a transmission-blocking vaccine, among others. Longer-term goals should include vaccine development, morbidity control, and suppression of transmission of these diseases as well as the development of better and more sensitive monitoring prior to and after chemotherapy (including those untreated/unvaccinated sections of the population in order to ascertain reductions in environmental transmission), and the improvement of hygiene, housing, and sanitation conditions, which will help with the sustainability of the achievements attained. A truly integrated management plan should therefore also include fundamental public health measures such as access to clean and potable water, adequate sanitation, waste disposal and improved environmental conditions, housing, and health education.

Moreover, as control programmes targeting a number of infectious diseases become integrated, co-formulation of pharmaceuticals could be advantageous. This will necessitate controlled studies of drug combination safety, and enhanced pharmacovigilance. In addition, if dose rates or formulations are changed to optimise antiparasitic efficacy, pharmacovigilance will become crucially important.

## The Need for Optimising Existing and Developing Novel Intervention Tools

### Optimisation of Existing Drugs

It is important to recognise that drug treatment regimes in helminth control strategies have generally not been optimised for treatment of human populations [45], let alone in integrated programmes for multiple NTDs, in which drug combinations need to be co-administered simultaneously or in staggered protocols. Very often, dose rates have been extrapolated from veterinary studies and treatment regimes have been designed to reduce the need for repeat treatment, or adopted following the dose rates used for other infections in drug donation programmes that expand their goals towards other infections. From the point of view of large-scale and community-directed anthelmintic delivery, as well as from the point of view of the companies manufacturing the pharmaceuticals, these may be important and practical considerations. However, as discussed above, sometimes the resultant drug efficacy is not optimal. This can have the consequences that the level of control/transmission interruption that is achieved is sub-optimal, and may increase selection pressure for drug resistance to develop. A related problem is that it is often difficult to assess accurately the efficacy of anthelmintics used against human parasites. Reliable biomarkers that could quantify parasite burdens could greatly assist with assessing efficacy, and this constitutes an important research challenge. Experiments involving parasite-animal models, followed by correlation studies between the biomarkers and existing means of assessing anthelmintic efficacy in humans, such as egg count reductions, would be required to validate such biomarkers.

As an example of the above mentioned problem with dose rates that are adopted from a drug donation programme for one disease to the expansion of such a programme to another disease, the dosage of IVM in the IVM + ABZ treatment used for LF control may be sub-optimal (the 150 µg of IVM per kg of body weight that is standard for onchocerciasis may not be the best for LF [45]). Optimisation of dose rates for these drugs will require additional research. In addition, yearly treatment has been followed for logistical reasons to attempt to achieve LF elimination. It is likely that more frequent treatment (6–9 months) with either DEC, DEC + ABZ, or IVM + ABZ would have a greater effect on suppression of transmission and shorten the time for elimination to be achieved (an area of research where mathematical modelling could assist greatly [78]). In addition, the advantages of more frequent treatment with ABZ (and IVM) on reducing morbidity due to STHs, as a collateral benefit of treatment for LF, may be greater than those achieved with annual treatment. With all of the helminth diseases of humans, there is a paucity of pharmaceuticals available, and this is of concern in view of the increasing deployment of large-scale mass administration programmes with the existing few drugs and the increased possibility of drug resistance development.

In the case of schistosomiasis, use of the levo-racemer allows a lower dose rate to be used, compared to the standard PZQ, which is a racemic mixture in which the d-PZQ has almost no effect [79]. However, little research has been undertaken on this topic in recent years.

### Anthelmintic Combinations

There are two reasons for considering the use of combination anthelmintics; firstly, to increase the spectrum, effectiveness, and convenience of drug administration, and secondly, to slow the development of resistance. In the first instance, two different anthelmintics might be given in order to integrate control programmes, e.g., schistosomiasis control with that of STH in school children, with PZQ and ABZ. The safety of combining ABZ + PZQ has been demonstrated [80], [81], and it is of interest that this combination increases the serum levels of ABZ sulphoxide. Alternatively, two anthelmintics may be given together (as separate medications or as a co-formulation) in order to increase the effectiveness of the drug treatment. Examples of this include the co-administration of ABZ with PZQ for neurocysticercosis, or ABZ with DEC (or IVM) for LF. In the latter example, the DEC (or IVM) exhibits microfilaricidal activity, has some limited adulticidal activity (in the case of DEC), and causes a temporary inhibition of reproduction by the adult parasites (IVM and DEC), while the ABZ extends the duration of inhibition of reproduction and exerts activity against some STHs as a collateral benefit. These extensions of the spectrum or effectiveness of chemotherapy may be desirable in their own right. Limited research has been done to assess whether combinations of different anthelmintics produce synergistic effects. Some evidence of synergy has been reported with combinations of artemisinins or synthetic peroxides with either PZQ or tribendimidine against *C. sinensis* [82], or with triclabendazole against *F. hepatica* [83]. Using the *Caenorhabditis elegans* model, significant synergy was reported between Cry proteins from *Bacillus thuringiensis* and the nicotinic acetylcholine receptor (nAChR) agonists tribendimidine and levamisole [84]. There is a need for more research to investigate whether combinations of different anthelmintics can bring significant synergies and improve control of helminth infections.

Combination chemotherapy is often suggested as a means of reducing selection of drug resistance. Drug resistance is recognised as a major hindrance to the control of many of the important infections, including malaria, tuberculosis, and HIV. Drug combinations, rather than monotherapy, are accepted as the best approach to treat all three diseases, with for example, the use of artemisinin-based drug combination now recommended by the WHO for malaria. The use of combinations to delay the development of anthelmintic resistance should be a research priority so that appropriate combinations are used and optimised for this purpose. In the treatment of veterinary parasites, the use of anthelmintic combinations to slow the development of resistance has been promoted in Australia and New Zealand [85], [86] but remains controversial [87], [88]. A number of conditions will determine whether combination chemotherapy will be better than monotherapy in slowing the development of resistance. Combinations will be most beneficial when: (i) resistance mechanisms, to the different anthelmintics in the combination, are under independent genetic control, i.e., do not share common mechanisms of resistance/resistance genes; (ii) resistance mechanisms are functionally recessive at the dose rates used; (iii) the genes for resistance are rare and the efficacy of each of the component compounds against the parasite population approaches 100%; (iv) a proportion of the population is not exposed to treatment, i.e., some *refugia* exist; and (v) the compounds used have similar half-lives [87]–[89].

However, at the current time, we may be far from these ideal situations in controlling helminth infections in humans. For example, some anthelmintics from different chemical classes may share similar resistance mechanisms and select on the same genes. There is a considerable literature indicating that ABC transporters are involved in IVM resistance (see [90] for review). However, benzimidazole anthelminthics may also select on some ABC transporter genes [91], [92]. Furthermore, changes in the isotype β-tubulin gene are known to cause benzimidazole resistance [93], while IVM also selects on the isotype 1 β-tubulin gene of some nematode parasites [74]–[76]. Thus, a combination of ABZ and IVM may be useful in terms of broadening the spectrum/efficacy of the combination, compared with each anthelmintic individually, but it may not reduce the rate of selection for resistance to one or both components of the combination. While there is no evidence that benzimidazole resistance causes cross-resistance to IVM, there is now some evidence that selection with IVM can select for benzimidazole resistance [76]. The other condition that appears to be problematic for the use of anthelmintic combinations for human helminth infections, in terms of delaying selection for resistance, is that the efficacy of current drugs used against human helminths is often not close to 100%. These examples highlight the importance of assessing any proposed combination against the criteria outlined above before recommending a combination as a means of delaying the selection for resistance. Nevertheless, there will likely be a role for combinations to expand spectrum or efficacy, as well as to provide increased convenience for MDA programmes. In addition, it is obvious that the co-administration of two anthelmintics should not result in increased side effects or toxicity. In this regard, it should be noted that some anthelmintics, such as IVM, are very potent inhibitors of ABC transporters [90] and could dramatically alter the pharmacokinetic behaviour, in the host and in the parasite, of other pharmaceuticals that are substrates for the same ABC transporters, and thus potentially increase toxicity to the host (undesirable) or to the parasite (desirable). Thus, research should be undertaken on possible interactions and selection on resistance mechanisms of possible combinations.

### New Drugs

There is a critical need for new anthelmintic drugs to be developed for human use. New anthelmintics for STHs, such as tribendimidine (which shares a mechanism of action with levamisole and pyrantel) [94], and nitazoxanide, do not appear to have superior efficacy against nematodes, compared with those commonly used and shown in Table 2. However, the opisthorchicidal activity of tribendimidine should be noted [95]. Research is required to assess the use of these new anthelmintics for MDA of STHs and possibly for food-borne trematodiases. The desirable characteristics for a new anthelmintic against STHs are outlined, as an example, in Table S1. Most of the existing anthelmintics used in human medicine were initially developed for parasite control in animals, where there is a significant commercial market. New veterinary anthelmintics (e.g., emodepside, derquantel) should be evaluated for potential use against helminth parasites of humans (see [2], [65]). Monepantel has been evaluated for its activity against STHs, but cannot be recommended [96]. In the current market-based system for drug development, registration, and marketing it is unlikely that pharmaceutical companies on their own will develop anthelmintics uniquely for human use. Nevertheless, pharmaceutical companies have been willing to work with public bodies and philanthropic foundations to develop anthelmintics for human use where the cost of such development is shared. In this way pharmaceutical companies can bring their considerable experience in drug discovery, screens, and chemical libraries to bear in the search for new anthelmintic molecules.

Moxidectin is under development for use in humans for the treatment of onchocerciasis [97]. Studies in model filarial infections and Phase 2 studies in individuals with *O. volvulus* indicate that it may result in a significantly greater suppression of microfilarial production by the adult parasites, maintaining the microfilaridermia at very low levels for up to 18 months. If this were confirmed in human clinical studies, treatment with moxidectin could be advantageous for onchocerciasis control compared with IVM. The development of IVM resistance in *O. volvulus* [98] could, however, possibly compromise moxidectin, as these two macrocyclic lactone anthelmintics appear to share (in veterinary nematodes) some, but not a complete set, of resistance mechanisms [99], and this requires investigation in populations in which there may be IVM-resistant parasites. If the apparent advantages of moxidectin for onchocerciasis control are confirmed, consideration should be given for its use against LF. In veterinary use, long-acting formulations of moxidectin have been developed and show significant therapeutic advantages against filarial parasites. Thus, long-acting formulations for human use should be considered if current development efforts with moxidectin prove successful. Not-for profit organisations such as the Drugs for Neglected Diseases *initiative* (DND*i*), with Michigan State and McGill University, have begun to evaluate flubendazole as a macrofilaricide for LF and onchocerciasis [100].

Another potential new drug development strategy is the possible use of antibiotics to target the *Wolbachia* endosymbionts in *O. volvulus* and *W. bancrofti*. However, at present the recommended antibiotic, doxycycline, seems unlikely to be suitable for large-scale mass chemotherapy due to the long course of daily treatment required to obtain desirable effects (6 weeks), and the fact that this drug is contraindicated in young children and pregnant or potentially pregnant women. Field trials have been conducted to test the feasibility of administering doxycycline via community distributors with good coverage and compliance in areas endemic for both onchocerciasis and loiasis [101]. A 6-week course of doxycycline has a marked macrofilaricidal effect. Given that *L. loa* is one of the filarial parasite species that lacks *Wolbachia* endosymboints, anti-*Wolbachia* therapy may be an attractive approach in those endemic areas where IVM cannot be used because of the possibility of life-threatening adverse reactions. Research is currently underway to identify alternative antibiotics that exert an anti-*Wolbachia* effect with a shorter course of treatment [102]. Continued efforts should be made to develop more effective antifilarial anthelmintics, and particularly to find drugs with macrofilaricidal activity.

### Novel Vaccines

Efforts to develop anti-helminth vaccines have gone on for many years and continue with steady progress in identifying candidate antigens, recently aided with the generation of a number of helminth genomes [103]. The successful TSOL18 porcine vaccine for cysticercosis [104] shows that it is indeed possible to create a highly effective vaccine against a multicellular parasite. Proof of concept of the feasibility of actively eliminating *T. solium* transmission in a wide endemic area of Peru and in Cameroon has been provided [7], [69,106]. Although it is very expensive to develop a vaccine for use in humans, in view of the very high efficacy and safety of the TSOL18 vaccine in animal models and the very serious morbidity caused by neurocysticercosis in humans, it would be desirable to assess the potential for using TSOL18 for prevention of *Taenia* infections in humans. For this, and other human helminth infections, efforts to increase vaccine efficiency and to provide long-lasting protection must continue. There have been some promising leads for the development of vaccines against human hookworm, onchocerciasis, schistosomiasis, and fascioliasis [107]–[113]. Particular attention is being paid to helminth proteases and animal model studies have shown promising results. In addition to efforts to develop vaccines that could be used for prophylaxis of helminthiases, current thinking includes using vaccines together with anthelmintics, as adjunct prophylaxis [114]. Such a strategy provides a novel revitalising concept in a field where control activities have remained exclusively focused on morbidity reduction using drug delivery [103]. The development of anti-helminthic vaccines that do not necessarily induce sterilising immunity could still reduce the likelihood of vaccinated individuals developing severe infections and thus reduce the burden of disease. Not only could vaccine-linked chemotherapy reduce overall morbidity, but it could also reduce rates of parasite infection and re-infection. This, in turn, could prolong the interval between repeated drug treatments and reduce the likelihood of selecting for anthelminthic resistance [115], [116].

## Monitoring and Evaluation

M&E should be an integral component of large-scale interventions, but is unfortunately sometimes inadequately pursued. When the intervention involves repeated use of anthelmintic drugs, the clinical and parasitological impacts of the treatments, including impacts on transmission of the parasite and possible adverse events, should be monitored to assess the effectiveness of the programme, as well as any possible side effects of repeated drug treatment, changes in compliance 7prevalence and intensity of infections are discussed in McCarthy et al. [117].

Whereas age-targeted chemotherapy programmes aim to control morbidity, parasite elimination will require prolonged mass treatment of all those infected at all levels of endemicity. This strategy will substantially shrink the size of susceptible parasite *refugia* (populations of untreated parasites, not subjected to chemotherapeutic pressure), thereby increasing selection pressure for anthelmintic resistance. It is important therefore that parasite elimination programmes that rely on chemotherapy put in place careful surveillance systems for prompt detection of transmission resurgence and monitoring of drug susceptibility.

### Monitoring for Emergence of Drug Resistance

Most helminth control programmes today concentrate heavily on anthelmintic treatment. Unfortunately, MDA programs are often less effective than originally planned and, depending on the initial disease prevalence, need to be continued for longer than initially anticipated, a situation that risks the development of drug resistance [118]. Examples of this need to extend MDA programmes include the extension of APOC from 2010 to 2015 after 15 years of operation, and the need to extend LF elimination programmes beyond the 4–6 years originally proposed [119]. Review of the Pacific LF Elimination Program [120] after 7 years of MDA with DEC + ABZ revealed that, while there had been successes, the goal of elimination had not been achieved and there was a need to extend the programme beyond the projected 2010 end date. In a study in south India [5], it was reported that despite 20 years of DEC-fortified salt and MDA with DEC, it is possible to identify individuals with microfilaraemia, albeit much reduced from pre-intervention levels. What is typically seen is a pattern of rapid decline in infection intensity levels, followed subsequently by a decline in prevalence levels in the first few years after commencement of the MDA programme. This pattern is well illustrated in the assessment of Global Programme to Eliminate Lymphatic Filariasis (GPELF) programmatic data available to the WHO from 20 countries and presented by Ottesen et al. [121], where it was reported that microfilaremia prevalence decreased dramatically with four rounds of MDA. However, beyond the fourth round of treatment, the rate of decline did not appear to be sustained and entered a relatively static period during which prevalence remained low, but did not approach zero as originally predicted. Furthermore, it is important to analyse such data at individual examples and not at averages, because it is in the outlier responses that signals of the potential development of drug resistance can be found.

The need to extend MDA programmes or to explain unsatisfactory results is often attributed to inadequate coverage (MDA programme factor), and this certainly can play a key role and does need examination. However, other explanations also should be considered, including the quality of the drug (pharmaceutical factors), the possibility that some individuals have compromised immunity (individual host factors), or the possibility that the parasite is not responding normally, such as is seen when drug resistance is selected or a particular parasite population is less responsive than populations seen elsewhere (tolerance or natural resistance in a sub-population of the parasite) (parasite factors). Where possible, all possible explanations should be examined, and some, such as coverage and response to treatment (possible resistance), should be monitored on an ongoing basis. In addition, the mathematical models that have been developed (see [78]) may need to be updated to reflect field experience after some years of efforts to eliminate helminth infections.

It is difficult to obtain reliable phenotypic evidence of anthelmintic resistance because of the mediocre efficacy of anthelmintics against *T. trichiura*, hookworms [3], *W. bancrofti*, and *O. volvulus* [45]; the fact that these parasites cannot be easily cultured in vitro under conditions that would mimic the in vivo effects of the drugs, or in animals that provide realistic models of infection in humans; the difficulties in sampling adequately for the target stages of the drugs, and because density-dependent processes may affect interpretation of post-treatment egg counts or skin/blood mf counts. In nematode parasites of farmed animals, there is a serious problem of anthelmintic resistance to all available anthelmintics that are used in humans (except DEC, which is rarely used in animals) [122]. Nevertheless, in parasites of veterinary importance, phenotypic evidence of resistance often goes undetected, and it has been estimated that the resistance level must reach at least 25% before resistance can be detected with conventional parasitological tests such as faecal egg count reduction tests for assessing anthelmintic efficacy against STHs [123]. This same insensitivity also exists with assessment of drug efficacy in human STHs. The possibility that anthelmintic resistance will develop in STHs, the limitations of current methods for its detection, and new biological and molecular assays for resistance have recently been reviewed [3].

There have been periodic indications of poor DEC efficacy against LF. Persistence of LF microfilaraemia has been reported in Haiti after DEC treatment [124], [125]. More recently, a sharp decline in all therapeutic indices with the mf count returning to pre-treatment levels by the fourth year of single annual DEC therapy was reported [126]. Even after 20 years of DEC, LF microfilaraemia may persist [6]. We do not have a test for possible DEC resistance and do not know whether these and other reports of unsatisfactory responses to MDA with DEC monotherapy could, in part, be due to DEC resistance. IVM resistance is now widespread in nematode parasites of farmed animals, and there is increasing evidence that it may have developed in some *O. volvulus* populations [104]. There is also recent evidence of IVM resistance emerging in the related filarial parasite *Dirofilaria immitis* in dogs [127]–[130]. So the possibility must be considered that IVM resistance could also develop in LF and should be monitored.

The persistence of *O. volvulus* skin mf, which could not be accounted for by new infections, after many treatments with IVM, has been reported [98], [131]. This is manifested as a greater ability of the adult female worms to recommence reproduction soon after IVM treatment than had been observed previously. There is a need to develop molecular marker(s) for IVM resistance in *O. volvulus* populations and to use such marker(s) to monitor, on an ongoing basis, for possible IVM resistance in areas under MDA with IVM.

The presence of schistosome populations that are apparently refractory to PZQ has been reported from time to time in different parts of the world [132]. Generally, 10%–20% (but the proportion may be higher in some cases) of infected patients will continue to excrete eggs after treatment. It was not understood to what degree this represented selection of a resistant population or incomplete elimination due to the presence of immature worms at the time of treatment. In a recent study, a population genetics approach was used to determine whether or not persistent *S. mansoni* were drawn from the same population as susceptible parasites [133]. The results showed that the persistent parasites were not selected by PZQ treatment, suggesting that they were not resistant parasites but were more likely to be derived from immature worms, at the time of treatment, which were not cleared by PZQ. Nevertheless, a persistent reduction in efficacy of PZQ in schistosomes would represent a grave threat to control programmes, as there is a paucity of alternative drugs, and monitoring for possible PZQ resistance in schistosomes should be undertaken as part of large-scale intervention programmes.

Triclabendazole resistance has become reasonably widespread in *F. hepatica* in livestock in developed countries [134] and it could occur in *Fasciola* infections in humans. This suggests that monitoring of treatment outcome is prudent in human fascioliasis.

## Elimination

Elimination of the parasites in a region provides the best and most sustainable outcome of intervention. Elimination of the infection reservoir is the goal of the GPELF and OEPA, and since the success story of suppression of *O. volvulus* transmission in the Oaxaca focus in Mexico [19] and in Mali and Senegal [20], it is also an objective for APOC. However, it does need to be recognised that despite, in many cases, 20 or 30 years of MDA, it has proven difficult to achieve elimination of onchocerciasis, STHs, schistosomiasis, and in some situations, LF. As discussed above, part of the problem is that the tools that we have available at present are inadequate to achieve elimination for all endemicity levels. Nevertheless, elimination should remain the ultimate goal of large-scale intervention efforts. Some of the critical issues that need to be addressed as we approach elimination are: (i) how to detect the presence of parasites and active transmission when infection prevalence and intensity are low; (ii) how to remove persistent low-level infections despite repeated treatment, and when drug resistance could be suspected; (iii) when to cease MDA; and (iv) for how long after apparent elimination should monitoring and surveillance continue. A discussion of the issues concerning diagnosis and monitoring can be found in McCarthy et al. [117], and the role of mathematical modelling in preparing for elimination in Basáñez et al. [78].

## Conclusions

The major multinational pharmaceutical companies provide many of the drugs used for mass treatment free of charge, or the drugs are available as low-cost generics, making the MDA programmes one of the most cost-effective global public health control measures. MDA programmes have led to major reductions in morbidity caused by helminth parasites. However, the sustainability of current interventions has been recently challenged by the difficulties in maintaining high coverage and compliance levels and the possible development of drug resistance. Moreover, the long-term control and elimination of these helminth diseases will also depend on improved control tools such as more effective anthelmintics and/or vaccines, control of vectors, intermediate hosts, or reservoirs, improved diagnostics and surveillance tools, sanitation, hygiene, socioeconomic improvement, and environmental sustainability of the interventions [135], [136]. If they become available, antiparasitic vaccines could have a major impact on sustained control of helminth diseases, and could be combined with MDA to provide complementary approaches that may improve control and reduce selection for drug resistance. Vector control, where applicable, practical and cost effective, remains a useful addition to chemotherapy-based intervention that can lead to marked reductions in transmission and reinfection rates. Efforts to integrate various MDA programmes may bring at first logistical challenges, but in the long-term benefits to the overall impact of intervention strategies. However, it should also be recognised that very little funding is available to support the necessary research. Key recommendations for research to improve intervention for control and elimination are summarised in Box 2 and a more detailed list of recommendations and gap analysis is available (see Text S1 for “Recommendations to Policy and Decision Makers: Gap Analysis and Identification of Research Priorities”). This research is necessary for interventions to remain sustainable and to achieve elimination of the helminth parasites that still bring suffering to millions.

**Box 2. Key Research Recommendations**

Successful intervention against human helminthiases depends on optimal utilisation of available control measures and development of new tools and strategies. This will require efforts to:

- **Optimise existing intervention tools** to maximise impact (including those against polyparasitic infections) and long-term sustainability. The tools include pharmaceuticals, vector control, vaccines, and eco-health approaches (sanitation, clean water, and increased food hygiene and education). Sustainability depends on minimising selection for drug resistance and maintaining community involvement and participation
- Develop strategies to **optimise the delivery, monitoring and evaluation** (M&E) of multiple interventions (drug combinations addressed at single or polyparasitic infections; combinations of antiparasitic and antivectorial measures) to various age groups, population levels (community, district), and geographical scales to maximise the effectiveness of integrated control
- **Develop novel control tools** that will improve impact and sustainability of the interventions, and increase the possibility of helminth elimination. These tools include new pharmaceuticals, the development of vaccines, innovative approaches to vector control, and an increased understanding of the environmental and social ecology of these infections
- **Minimise the development of anthelmintic resistance**, and enhance preparedness. This will require research and development to:
  1. Develop assays that can be used to promptly detect changes in intervention efficacy and investigate the association of such changes with parasite genetic and non-genetic factors
  2. Develop sensitive and efficient tools for resistance monitoring that can be applied in disease-endemic countries in operational research settings by control teams, and be integrated into intervention programmes as part of M&E

## Supporting Information

Table S1 Desirable Characteristics for a New Anthelmintic for Soil-Transmitted Helminths (STHs).
(DOC)

Text S1 Recommendations to Policy and Decision Makers: Gap Analysis and Identification of Research Priorities.
(DOC)
