# Supplementary material for: A Research Agenda for Helminth Diseases of Humans: Intervention for Control and Elimination
Source: PLoS Negl Trop Dis. 2012 Apr 24;6(4):e1549. doi: 10.1371/journal.pntd.0001549 (PMC3335868; doi:10.1371/journal.pntd.0001549)
Supplement: Table S1 — Desirable Characteristics for a New Anthelmintic for Soil-Transmitted Helminths (STHs). (DOC) [file pntd.0001549.s001.doc]

**Supplementary Table 1. Desirable characteristics for a new anthelmintic for soil transmitted helminths (STHs)** (Modified from [2])

| **Property** | **Desirable (Minimal) Product Characteristics** | **Added Value Product Characteristics** |
| --- | --- | --- |
| **Route of administration** | **Oral (essential)** |  |
| **Activity against all species of human STH** | Active at least against adult stages of principal geohelminths, including *Ascaris*, hookworms (*Ancylostoma and Necator*), *Trichuris*, *Enterobius* and Strongyloides at the target dose. | Additionally active against cestodes and/or trematodes in multiple doses (<=3 days) or at higher single doses  Active against tissue migrating stages and systemic helminth species with short courses of treatment (1-3 days) |
| **Activity against all stages in humans** | Active against lumen dwelling adults.  Ovicidal. | Active against migrating larvae and tissue stages of STHs. Active against immature stages of cestodes in tissues. |
| **Active against resistant organisms** | Not sharing same mechanisms of resistance (mode of action receptor resistance or resistance due to common transport/metabolism mechanisms), Likely to be novel molecular class. | Drug with low potential for inducing resistance. |
| **Dosing schedule** | **Single dose (preferable) or maximum two doses in one day (essential) against main STNs (*Ascaris*, hookworms, *Trichuris*)** |  |
| **Clinical safety** | Safety profile includes long term safety and mild side effects (not worse than existing agents), side effects in uninfected individuals minimal | Safety profile that permits use in Mass Drug Administration control programmes; without contraindications with concurrent other drug administration or concurrent non-target infections |
| **Clinical efficacy** | **High efficacy: >95% cure (>95% egg reduction) of *Ascaris* and both Hookworm infections; >90% cure (>90% egg reduction) of Trichuris infections** |  |
| **Clinical use** | Safe for administration without medical supervision – especially if given without screening. |  |
| **Compatibility with potential partner drugs** | Concomitant treatment with ivermectin, praziquantel or benzimidazole anthelminthics. Possible to partner to improve spectrum or cure rates or reduce risk of resistance. | Capable of integration into multiple drug treatment programmes |
| **Drug-drug Interactions** | No interaction with ivermectin, benzimidazoles, praziquantel or drugs for malaria or HIV/AIDS |  |
| **Use in pregnant/lactating women** | Safe during trimester 2 and 3 of pregnancy and during lactation  No teratogenetic signals in toxicology | Safe during all trimesters of pregnancy |
| **Use in infants and children** | Safe for use **from 1 year of age;**  Specific oral tablet **or suspension** for small children | Simple dosing schedule based on weight or height. |
| **Cost per treatment** | **Affordable at point of use**, therefore equivalent to cost of current treatments |  |
| **Quality** | Good Manufacturing Practices (GMP) quality | Process capable of transfer to provide endemic country sourcing |
| **Storage requirements** | Two years shelf life at high humidity and temperatures (tropical standard – (40^o^ C/75%RH) | Shelf life > 2 years (extended real time) |
| **Other characteristics** |  | Also active against other helminth diseases – onchocerciasis, lymphatic filariasis and schistosomiasis.  Can administer one standard dosage for adults and children, or use height (not weight) as a determinant of dosage in children |
