# Supplementary material for: A Research Agenda for Helminth Diseases of Humans: Intervention for Control and Elimination
Source: PLoS Negl Trop Dis. 2012 Apr 24;6(4):e1549. doi: 10.1371/journal.pntd.0001549 (PMC3335868; doi:10.1371/journal.pntd.0001549)
Supplement: Text S1 — Recommendations to Policy and Decision Makers: Gap Analysis and Identification of Research Priorities. (DOC) [file pntd.0001549.s002.doc]

**Supplementary Text S1**

**Recommendations to Policy and Decision Makers: Gap Analysis and Identification of Research Priorities**

Successful intervention against human helminthiases depends on optimal utilization of available control measures and development of new tools and strategies. This will require to:

- 1. Optimize existing control methods to reduce the duration of control / elimination programmes and reduce possible selection for drug resistance:

i Pharmaceuticals, in single and combination therapies

ii Paediatric formulations of ivermectin and praziquantel

iii Vector control, particularly in combination with MDA

- - 1. Develop novel helminth control methods to treat infection and reduce transmission:

i Pharmaceuticals

ii Vaccines, particularly when combined with MDA

iii New vector control tools

- - 1. Minimize development of resistance and enhance preparedness (this includes resistance to anti-parasitic and anti-vectorial interventions):

i Develop parasitological assays that can be used for prompt detection of changes in intervention efficacy and investigate the association of such apparent changes with parasite genetic and non-genetic factors (e.g. changes in frequencies of putative alleles under selection; poor coverage / adherence; intense transmission)

ii Based on the knowledge derived from the aspects investigated in 3.i, develop sensitive and efficient tools for resistance monitoring that can be applied in disease endemic countries by operational research and control teams

iii Once appropriate markers have been identified, integrate their use into M&E and surveillance activities to ensure routine monitoring for resistance

iv Develop strategies to reduce the spread of resistant parasites (e.g. vaccine linked chemotheraphy, vector control)

- - 1. Address polyparasitism:

i Develop improved systems to integrate the delivery and the M&E of multiple strategies for the simultaneous control of co-endemic infections

ii Implement systems for pharmacovigilance of the pharmaceutical combinations that may be needed for 4.i as well as for new dose rates or formulations

iii Understand and prevent/address IVM-related SAEs in some *Loa loa* hyperendemic areas

Currently, many of the ongoing anthelmintic control programmes are based on annual MDA. While this has logistical advantages and seems to have worked well in morbidity reduction programmes based on preventative chemotherapy (e.g. schistosomiasis, onchocerciasis), annual distribution may not necessarily be optimal to achieve transmission control or local elimination of infection. By the same token, it may be necessary to decrease treatment frequency in areas of low transmission according to cost-effectiveness studies. Therefore, research is needed to:

- - 1. Assess the benefits of semi-annual versus annual treatment for onchocerciasis and LF, particularly in Africa (in the Americas onchocerciasis programmes already treat twice per year), as well as the benefits of reducing treatment frequency from annual to biennial (e.g. in hypoendemic schistosomiasis areas) in terms of:

i Reductions of morbidity, transmission, and timelines to elimination

ii Cost-effectiveness of interventions (in terms of the number of infections, sequelae, and excess deaths averted. Implement systems for pharmacovigilance of the pharmaceutical combinations that may be needed for 4.i as well as for new dose rates or formulations

iii Possible positive or negative implications for selection of drug resistance

**6.** Determine how new control tools that may become available should be used to optimize the impact of the interventions. For instance, in the filariases research is required for:

i Moxidectin. How should it be used for onchocerciasis and LF: in ivermectin-naïve areas; when sub-optimal responses to ivermectin have been characterized; as an alternative to conventional ivermectin use, but possibly aiming to suppression of transmission rather than morbidity control.

ii Anti-*Wolbachia* chemotherapy. How should it be used for onchocerciasis and LF: in the modality of MDA; for mop-up operations; in combination with IVM; in areas showing sub-optimal responses to ivermectin; or on its own in areas co-endemic for onchocerciasis and loiasis? Can duration of treatment be considerably shortened from the current regimes of 4–6 weeks of daily treatment with doxycycline?

**7.** Develop and validate guidelines for stopping MDA in different settings with different approaches
